# Functional Connectivity Analyses in Imaging Genetics: Considerations on Methods and Data Interpretation

**DOI:** 10.1371/journal.pone.0026354

**Published:** 2011-12-29

**Authors:** Johannes Bedenbender, Frieder M. Paulus, Sören Krach, Martin Pyka, Jens Sommer, Axel Krug, Stephanie H. Witt, Marcella Rietschel, Davide Laneri, Tilo Kircher, Andreas Jansen

**Affiliations:** 1 Department of Psychiatry and Psychotherapy, Philipps-University Marburg, Marburg, Germany; 2 Department of Neurology, Philipps-University Marburg, Marburg, Germany; 3 Department of Genetic Epidemiology in Psychiatry, Central Institute of Mental Health, Mannheim, Germany; Beijing Normal University, Beijing, China

## Abstract

Functional magnetic resonance imaging (fMRI) can be combined with genotype assessment to identify brain systems that mediate genetic vulnerability to mental disorders (“imaging genetics”). A data analysis approach that is widely applied is “functional connectivity”. In this approach, the temporal correlation between the fMRI signal from a pre-defined brain region (the so-called “seed point”) and other brain voxels is determined. In this technical note, we show how the choice of freely selectable data analysis parameters strongly influences the assessment of the genetic modulation of connectivity features. In our data analysis we exemplarily focus on three methodological parameters: (i) seed voxel selection, (ii) noise reduction algorithms, and (iii) use of additional second level covariates. Our results show that even small variations in the implementation of a functional connectivity analysis can have an impact on the connectivity pattern that is as strong as the potential modulation by genetic allele variants. Some effects of genetic variation can only be found for one specific implementation of the connectivity analysis. A reoccurring difficulty in the field of psychiatric genetics is the non-replication of initially promising findings, partly caused by the small effects of single genes. The replication of imaging genetic results is therefore crucial for the long-term assessment of genetic effects on neural connectivity parameters. For a meaningful comparison of imaging genetics studies however, it is therefore necessary to provide more details on specific methodological parameters (e.g., seed voxel distribution) and to give information how robust effects are across the choice of methodological parameters.

## Introduction

Imaging genetics combines genotype assessment with neuroimaging to identify structural or functional brain systems that mediate genetic vulnerability or liability to mental disorders [1]. Due to the proximity to the genetic level, in this intermediate phenotype approach the penetrance of genetic variation is regarded to be higher on the neural systems level than on the level of emergent mental or behavioral phenomena [2], [3]. Technically, imaging genetics combines neuroimaging data and genetic risk variants using random-effects analyses. For functional imaging data, it has been proposed that in particular the connectivity features of neural systems are associated to genetic risk to a higher degree than regional activation data [4].

Using functional magnetic resonance imaging (fMRI), connectivity aspects of brain systems can be described in several ways. One approach that is frequently applied in imaging genetics studies is functional connectivity, defined as the “temporal correlations between spatially remote neurophysiological events” [5]. Currently, two analysis approaches are predominantly used to examine functional connectivity with fMRI data: seed voxel correlation analysis and independent component analysis (for an overview see [6], [7], [8]). Studies in imaging genetics predominantly used a seed voxel approach (e.g. [4], [9], [10], [11]). Thereby, the fMRI signal time course is extracted from a region-of-interest, the so-called “seed region”. Then, the temporal correlation between the extracted signal and the signals from all other voxels in the brain volume is determined. This procedure can be both applied to data acquired during rest and data acquired under controlled experimental conditions. In the latter, task effects are typically removed through an appropriate statistical model, that is, regional activation related to the task is subtracted from the data. Most studies in imaging genetics so far used data acquired during experimental conditions (e.g. [12], [13], [14]).

FMRI data is contaminated by various fluctuations unrelated to neural activity, for instance residual subject motion, physiological artifacts, hardware instabilities and magnetic field drifts [6], [8], [15]. These non-neural signal fluctuations may introduce temporal coherences that cause an overestimation of functional connectivity between remote brain regions. Thus, confounding effects have to be removed before connectivity assessment and comparison between groups to maximize the variance components of genetic risk associated neural systems phenotypes in the total variance observed between subjects. Several studies, however, showed that the “preprocessing” of fMRI data has a substantial impact on the resulting connectivity pattern. The results of seed voxel connectivity analyses are sensitive, for instance, to seed selection [16], [17] and noise reduction algorithms [15], [18].

The application of functional connectivity methods to imaging genetics provided evidence for genetic control over connectivity features of neural systems. Many of the obtained results though still have to be replicated in independent samples. Reliability of fMRI data, however, is influenced by many factors, among others by the specific implementation of data analysis procedures [19] thus rendering the replication of promising findings a non trivial endeavor. Using the examples of seed voxel selection, noise correction algorithms, and inclusion of seed voxel localization as covariates on the group level, we demonstrate in the present report that the choices of freely selectable parameters during data analyses strongly influence the assessment of the genetic modulation of functional connectivity features. Rather than presenting new evidence for intermediate phenotypes of a specific genetic risk variant, with this proof-of-principle study we show that for meaningful comparisons of studies in imaging genetics comprehensive information about the analysis process should be presented and the impact of arbitrary decisions during the data analysis on the result and their interpretation should be considered.

## Methods

### Ethics Statement

We confirm that the research has been conducted in compliance with the appropriate ethical guidelines of the declaration of Helsinki. The study was approved by the local ethics committee at the faculty of medicine, University of Aachen. All subjects were written informed about the background of the study and anonymity of data collection. We confirm that we obtained informed written consent from all participants involved in the study.

### Subjects

As part of a study on the genetic basis of schizophrenia and bipolar disorder, 94 healthy subjects were included in the present analysis. Inclusion criteria were age (18–55 years), right-handedness (as assessed by the Edinburgh Inventory, [20]), no psychiatric disorders according to ICD-10, no family history of schizophrenia or bipolar disorder, and Western- or Middle European descent. In the present study, we exemplarily focus on the modulatory effects of the single nucleotide polymorphism (SNP) rs1006737 of the *CACNA1C* (alpha 1C subunit of the L-type voltage-gated calcium channel) gene, a susceptibility locus for both bipolar disorder and schizophrenia [21], on the neural correlates of working memory. Subjects were divided into three groups according to their rs1006737 genotype (Table 1). A description of the genetic analysis can be found elsewhere [22].

**Table 1:** Sample characteristics: sex, age and education. According to their rs1006737 genotype subjects were divided into three groups (G/G, G/A, and A/A). G = Guanine, A = Adenine (risk allele).

rs1006737 genotype	G/G	G/A	A/A
number of subjects	44	38	12
Sex ratio (men/women)	36/8	24/14	6/6
Age (years)	23.5±3.3	23.1±2.8	23.0±1.0
Education (years)	15.9±2.8	15.4±2.5	15.8±1.6

### FMRI paradigm

Working memory was assessed by a letter variant of the n-back task. A detailed description of the paradigm can be found elsewhere [23]. In short, the paradigm consisted of three conditions: letter fixation as a high-level baseline, 0-back, and 2-back. In each condition sequences of Latin letters were presented. During 0-back, responses were required for the target letter “X” and in the 2-back condition, target letters were defined as all letters which were identical to the one presented two steps before. Four 0-back blocks (selective attention) were alternated with four 2-back blocks (working memory) and with eight baseline blocks (letter fixation) in between conditions. Each condition was preceded by instructions explaining the respective task.

### MRI data acquisition

Data was acquired on a 3 Tesla TIM-Trio MR scanner (Siemens Medical Systems) at the Forschungszentrum Jülich. Functional images were collected with a T2*-weighted echo planar imaging (EPI) sequence sensitive to BOLD contrast (64x64 matrix, FOV 200 mm, in plane resolution 3.13 mm, 36 slices, slice thickness 3 mm, TR = 2.25 s, TE = 30 ms, flip angle 90°). Slices covered the whole brain and were positioned transaxially parallel to the anterior-posterior commissural line. Two hundred and seventeen functional images were collected, and the initial three images were excluded from further analysis to remove the influence of T1 saturation effects.

### FMRI data analyses

SPM5 (www.fil.ion.ucl.ac.uk/spm) standard routines and templates were used for the analysis of the fMRI data. After slice-timing, functional images were realigned, normalized (resulting voxel size 2x2x2 mm^3^), smoothed (8 mm isotropic Gaussian filter) and high-pass filtered (cut off period 128 s). After preprocessing, a quality control of each subjects' images was implemented to assure that results of the following functional connectivity analyses are not atypically influenced by head motion. Movement parameters of all subjects were in an acceptable range not exceeding 3 mm for each single subject. Maximum translations and rotations as well as the averages and standard deviations of the subjects' movement parameters within a session did not vary with rs1006737 genotype in an ANOVA, F<1.45, ps<.240. To determine brain activity, statistical analysis was performed in a two-level, mixed-effects procedure. At the individual subject level, a fixed-effects general linear model (GLM) included three epoch regressors, modelling the 2-back condition, the 0-back condition, and the instructions, as well as six regressors modelling head movement parameters. Parameter estimate (*ß*-) and *t*-statistic images were calculated for each subject. At the group level, weighted *ß*-maps of each subject describing activation differences between the 2-back and the 0-back condition were entered into a random-effects GLM with rs1006737 genotype (G/G, G/A, A/A) as between subject factor. Since sex was unequally represented in each group we included sex as covariate of no interest.

The aim of the present work was to describe the impact of different implementations of seed region functional connectivity analyses on the association of genetic variation with brain connectivity patterns. In the following, we will first describe one way how to implement a functional connectivity analysis (“standard implementation”). Then, we describe three variations of the analysis procedure with regard to (i) seed voxel selection, (ii) implementation of noise regressors, and (iii) the use of individual seed voxel coordinates as additional covariates. We will show how these variations influence the effects of genetic risk factors on the functional connectivity pattern.

#### Analysis of functional connectivity (“standard implementation”)

**A**s seed region we chose the right dorsolateral prefrontal cortex (DLPFC). For each subject, we selected individual seed voxels within the DLPFC to account for interindividual differences in functional activation patterns. Starting at the DLPFC peak activation on the group level at (52, 30, 30), we identified the next local maximum within each subject for the 2-back vs 0-back contrast at *p*<.01 uncorrected. To ensure that the extracted time series were located within the right DLPFC we applied a DLPFC mask defined by Brodmann area (BA) 9 and the lateral sections of BA 46 (Wake Forest University, WFU, PickAtlas software, www.fmri.wfubmc.edu). Additionally, we limited the next local maximum procedure to clusters extending 20 voxels. Seed time series were extracted as the first eigenvariate in a sphere of 6 mm radius as implemented in SPM5. Task related variance was removed by applying an effects-of-interest correction with the F-contrast set on the six movement parameters. Statistical analysis was performed in a two-level, mixed-effects procedure. At the individual subject level, the fixed-effects GLM included the extracted seed time series from the right DLPFC, two regressors for the 2-back and 0-back conditions, one regressor modelling the instruction period and six regressors modelling head movement parameters. To account for noise, two additional noise regressors were created by extracting time series for each subject from the first eigenvariates of masks covering medial cerebrospinal fluid regions (CSF) or white matter (WM). For extraction of the noise time series results were not thresholded (*p*<.99). Parameters of the GLM were calculated for each subject and *ß*-maps of the seed time series (*connectivity maps*) were analysed on the second level. On the second level, we conducted region-of-interest (ROI) analyses for the bilateral DLPFC and the bilateral hippocampal formation (HF). All statistical maps were thresholded at *p* = 0.05, corrected for multiple comparisons applying the family-wise error (FWE) correction implemented in SPM5.

#### Analysis 1: Influence of seed voxel selection

**T**here are several possibilities to select seed voxels in the right DLPFC. In the “standard implementation”, we started at the group activation maximum in the right DLPFC and identified for each subject in the first level activation maps the next local maximum. Another algorithm to select individual seed voxels is searching in every subject's first level activation map (2-back>0-back) for the global maximum within the DLPFC mask at p = 0.01, uncorrected (e.g. [9]). We compared the effect of different implementations of seed voxel selection on the results of genetic analyses using a second level 2x3 ANOVA model with the factors method (next local maximum vs. global maximum) and rs1006737 genotype (G/G, G/A, A/A). Sex was included as covariate of no interest. We analysed the interaction between method and rs1006737 genotype to assess regions in which a possible effect of genotype is indicated differently by both methods.

#### Analysis 2: Influence of the implementation of different noise regressors

**S**ince signal fluctuations from non-neural processes can cause an overestimation of functional connectivity, it is important to remove confounding effects prior to connectivity assessment. In the “standard implementation” of the connectivity analysis, we included noise regressors derived from time courses in WM and CSF. In this analysis, we assessed which effect the additional implementation of a global normalization procedure has on the results of genetic modulation. Global normalization, as implemented in SPM5, was performed by image-wise multiplicative intensity normalization. In every image, each voxel was divided by the mean intensity over all voxels and multiplied by the mean intensity of the first image. We compared the effect of different implementations of noise regressors on the results of genetic analyses using a second level 2x3 ANOVA model with the factors method (without global normalization vs with global normalization) and rs1006737 genotype (G/G, G/A, A/A). Sex was included as covariate of no interest. We analysed the interaction between method and rs1006737 genotype to assess regions in which a possible effect of genotype is indicated differently between both methods.

#### Analysis 3: Influence of seed voxel coordinates as covariates on the group level

**B**oth on the first and the second level, there are various possibilities to include additional covariates that account for confounding effects, for instance to correct for non-neural effects (e.g. scanner instabilities) or for interindividual differences between subjects (e.g. volume of specific brain regions). In this analysis, we assessed the effect of the additional inclusion of individual seed voxel coordinates as covariates of no interest in a second level linear regression model coding an additive gene-dosage effect of rs1006737 genotype (G/G<G/A<A/A). Sex was also included as covariate of no interest in both models.

## Results and Discussion

In the following we first present the results of the three analyses in which we exemplarily demonstrate the influence of different implementations of functional connectivity analyses on the assessment of genetic effects. Then, we discuss the implications of this methodological variability on imaging genetics studies. We will argue that researchers have to make explicitly clear why they make specific choices in the analysis process and which impact these choices have on the assessment of genotype effects.

### Analysis 1: Influence of seed voxel selection

**Figure 1:**
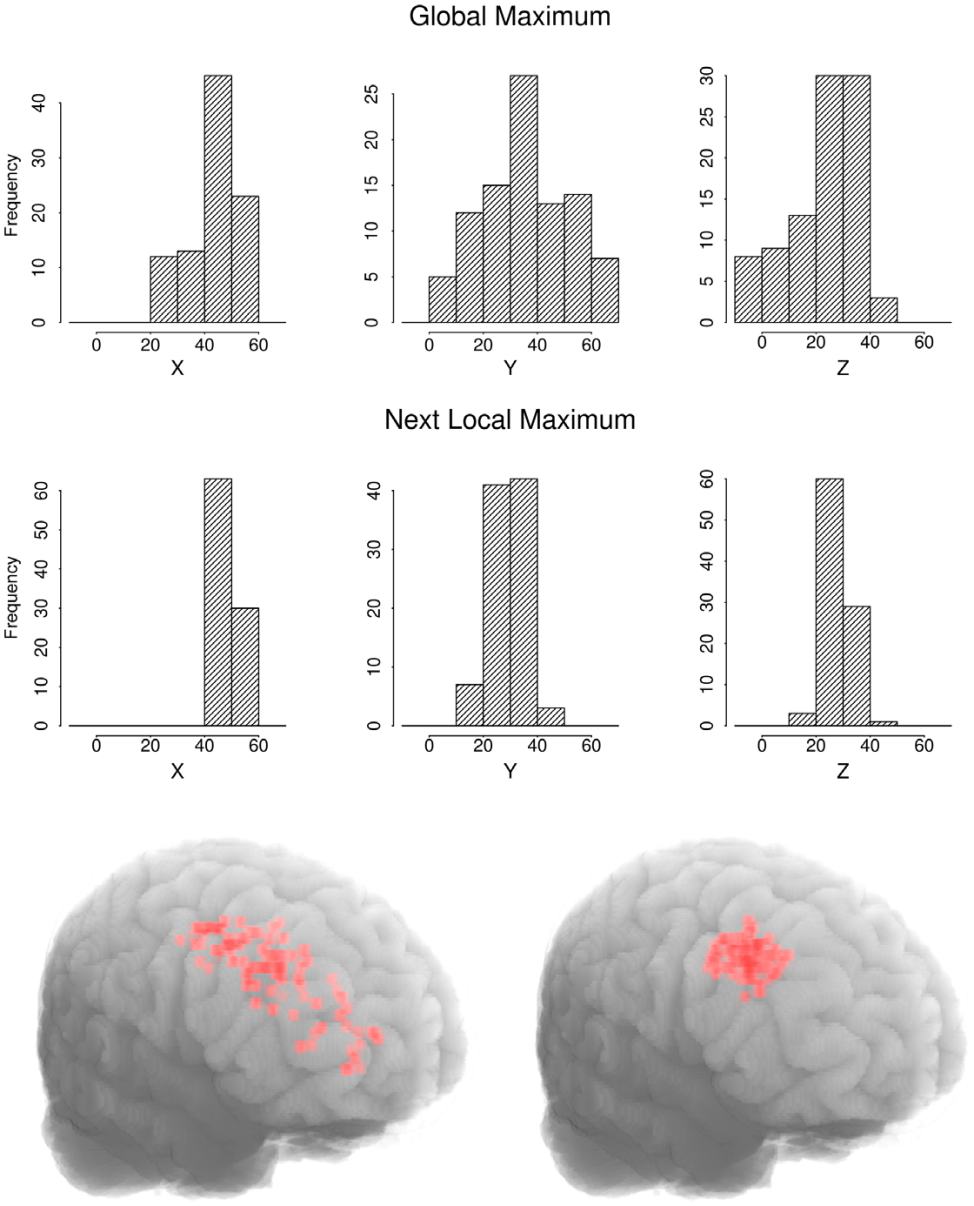
Seed Voxel Localization. Top: Frequencies of MNI-coordinates in the X, Y, and Z dimension for right DLPFC seed regions identified with the global maximum and the next local maximum starting at 52, 30, 30. Bottom: Individual seed-region localization in the right DLPFC for the functional connectivity analyses based on the global maximum (left) and the next local maximum approach (right).

The spatial distribution of the DLPFC seed regions is depicted in Fig. 1. The seed points from the global maximum implementation varied largely in their location along the y- and z-axes. A k-means cluster analysis with the x, y, and z MNI coordinates as spatial input variables showed that the seed points were clustered around two centroids located in the posterior (MNI coordinates 48, 29, 31) and anterior DLPFC (MNI coordinates 35, 56, 7), with a total Euclidian distance of 3.8 cm between both clusters. While the posterior cluster was located at the strongest DLPFC group activation maximum, the anterior cluster corresponded to another local activation maximum within the right DLPFC (at MNI coordinates 34, 58, 10). In contrast, the seed points identified with the next local maximum approach in the “standard implementation” were more homogeneous. In particular, the seed points did not originate from different local activation maxima.

**Figure 2:**
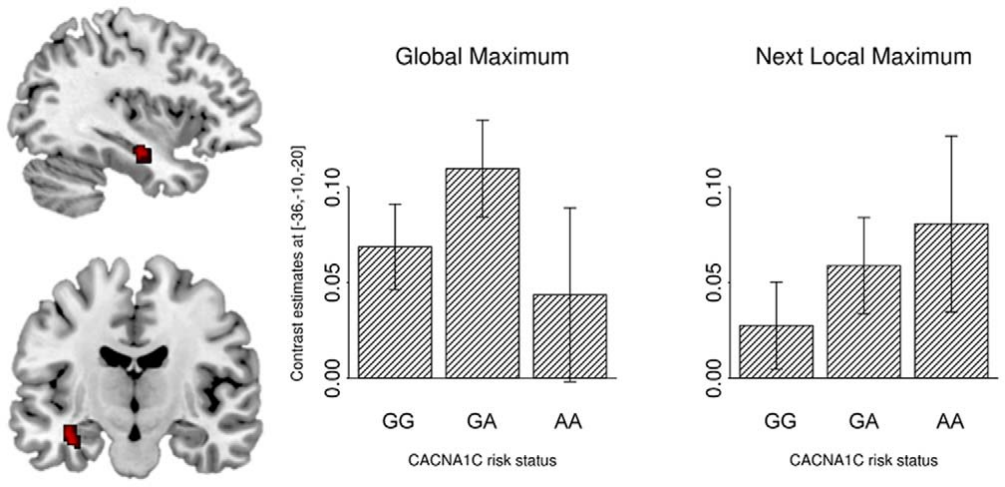
Influence of Seed Voxel Localization on the Comparisons of Genetic Groups. Left: Interaction between the factors method (next local maximum vs. global maximum) and rs1006737 genotype (G/G, G/A, A/A) in the left anterior HF. Right: A post-hoc analysis of the parameter estimates of each risk group separately for both methods. A significant linear gene-dosage effect is found only with one method of seed region selection, the next local maximum approach.

We found a significant interaction of method and rs1006737 genotype in the left anterior HF (MNI coordinates −36, −10, −20; Z = 4.41; p_cor_ = 0.002) (Fig. 2). The analysis of the parameter estimates of rs1006737 genotype groups for each method showed that the interaction occurred because one method (the next local maximum approach in the “standard implementation”) showed a linear gene-dosage effect, supporting the fronto-hippocampal dysconnectivity hypothesis of schizophrenia [24], while the other method (the global maximum approach) did not. (Of note, the term “dysconnectivity” also encompasses *increased* functional coupling between brain regions, in contrast to the term “disconnectivity” which only implies decreased coupling). These results demonstrate that different procedures to select seed voxels do not only effect the seed voxel distributions but significantly influence the subsequent analyses on the group level.

The seed points identified with the global maximum approach belonged to two different activation maxima within the right DLPFC possibly representing different functions. For our data the next local maximum method is therefore the more appropriate implementation of a seed-based connectivity analysis. However, for other data sets the global maximum approach yielded similar seed distributions that we obtained with the next local maximum method [23]. It is thus not possible to give a general advice which method of seed voxel selection is most appropriate under any condition. However, since the seed voxel selection has an impact on the results of subsequent group comparisons, imaging studies should present, at least as supplementary information, the spatial distribution of their seed voxels. This allows for a better comparison between studies and helps to understand whether conflicting findings result from methodological differences in the implementation of the connectivity analysis or are attributed to variability of genetic effects or sampling error.

### Analysis 2: Implementation of noise regressors

**Figure 3:**
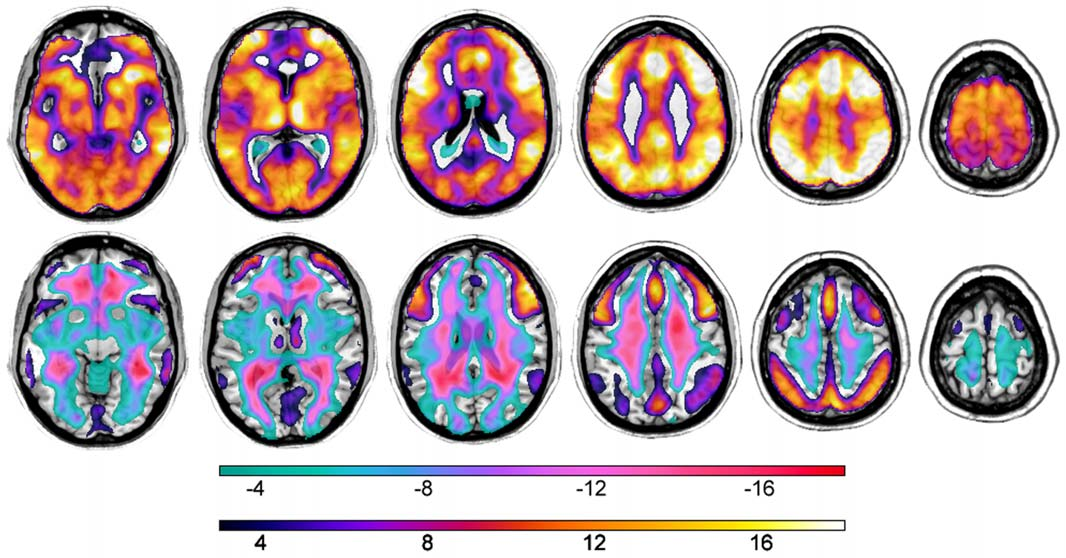
Influence of Noise Regression Methods on Connectivity Patterns. Functional connectivity group results (using one sample t-tests) with respect to the right DLPFC for the “standard implementation” of the connectivity analysis (top) and the additional global signal correction by application of the global normalization procedure (bottom). Yellow-red: positive correlations with seed voxel, green-blue: negative correlations with seed voxel.

The average functional connectivity patterns of the two preprocessing approaches, the “standard implementation” without global normalization and the one with additional global normalization are shown in Fig. 3. Both correlation profiles illustrate the functional integration of the network activated during the working memory task even after removal of task related variance. Strongest association with the seed time series emerged in the bilateral DLPFC, superior medial frontal cortex, the inferior parietal lobule, basal ganglia, and the cerebellar hemispheres. Without global normalization most time-series in cortical as well as subcortical brain regions were positively correlated with the right DLPFC, indicating common noise of seed and target time series still present after inclusion of WM and CSF regressors to control for noise. The global normalization reduced common noise and increased the specificity of the functional connectivity pattern. Accordingly, brain regions deactivated during 2-back, the ventromedial prefrontal cortex and middle temporal areas, while still positively associated with the DLPFC in the “standard implementation”, showed negative correlations only after global normalization on the first level.

**Figure 4:**
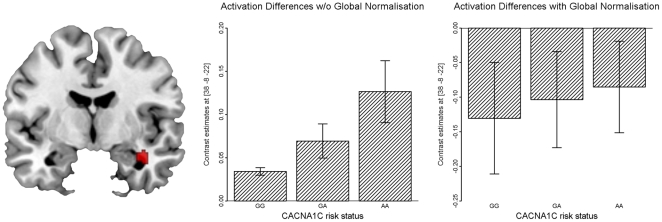
Influence of Noise Regression Methods on the Comparisons of Genetic Groups. A linear increase with with rs1006737 genotype in functional coupling between the right DLPFC (seed voxel) and the right hippocampus is detected for both noise reduction algorithms. However, the interpretation of the changes depending on the underlying correlation profile. While in the “standard implementation” one would argue that the hippocampus is decoupled in GG carriers and that coupling increases with genetic risk (GG<GA<AA), after global normalization the results suggest that medial temporal areas are anticorrelated with the DLPFC and at increasing genetic risk this negative coupling is downregulated.

Even though we did not find a significant interaction of preprocessing method and rs1006737 genotype, the different noise regressors influence the statistics of the subsequent group comparison insofar as results become significant using one procedure but not the other (Fig. 4). Even more important, the interpretation of the results obtained in the random-effects analyses changes according to the underlying correlation profile. For example, in both analyses we found a linear increase of connectivity with increasing genetic risk. However, while in the “standard implementation” one would argue that the medial temporal area is decoupled in GG carriers and that coupling increases with genetic risk (GG<GA<AA), after global normalization the results suggest a different interpretation. Medial temporal areas are anticorrelated with the DLPFC and at increasing genetic risk this negative coupling is downregulated to zero. Both interpretations might be valid, however, one should consider the impact of different noise reduction algorithms on the obtained results. While the “standard implementation” procedure results in artificial increased connectivity due to the influence of uncontrolled common noise, global normalization introduces artificial anti-correlations in the data (see also [18]).

### Analysis 3: Influence of seed voxel coordinates as covariates on the group level

**Figure 5:**
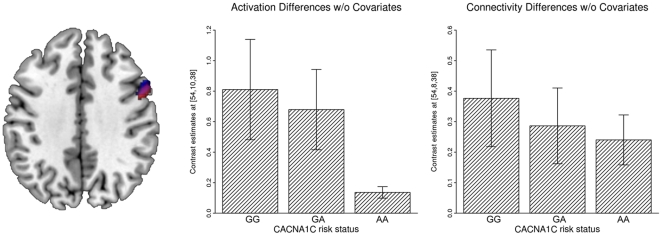
Influence of Seed Voxel Coordinates as Covariates on the Group Level. Reduction of genotype effects after entering seed-voxel localization as covariate on the second level. Left: A linear decrease in regional activation is found with number rs1006737 risk alleles in the right DLPFC (blue). A similar gene-dosage dependent effect in functional connectivity was found at the same location (red). After controlling for differences in the spatial distribution of seed-voxel localization with introduction of the MNI coordinates in the x-, y-, and z-dimension as covariate on the second level the associations of genotype with DLPFC connectivity was reduced and non-significant at p>.05 corrected

Coding an additive gene-dosage effect of rs1006737 genotype in a linear regression model, we found a significant linear decrease in *regional brain activation* in the right posterior DLPFC with increasing number of risk alleles (MNI coordinates 54, 10, 38; Z = 4.02; p_cor_ = 0.019, see Fig. 5). In the “standard implementation” of the *functional connectivity analysis* we found, at the same location, a significant decrease in functional coupling with the DLPFC seed region in rs1006737 risk-allele carriers (MNI coordinate 54, 8, 38; Z = 3.89; p_cor_ = 0.035). The most obvious interpretation might be that hypoactivity in the posterior DLPFC is caused by hypoconnectivity to core regions of the working memory network, that is, to the strongest activation cluster in the DLPFC. Both analyses, the analysis of regional activation and functional connectivity, seem to give complementary results, relating hypoactivity to dysconnectivity within the right DLPFC.

Although the seed voxel selection method based on the next local maximum approach yielded a relatively homogeneous distribution, a post-hoc analysis of the spatial distribution of the seed voxels in the three rs1006737 genotype groups showed a trend for differences in seed region localizations along the y-dimension at different rs1006737 genotype (ANOVA, F(2, 90) = 2.96, p = .057), which was significant if tested for a linear association with rs1006737 genotype (GG<GA<AA, r = .24, p = .023). After we additionally included the MNI-coordinates of the individual seed voxels in the x-, y-, and z-dimensions as covariate of no interest in the linear regression model we did not find a significant association of rs1006737 genotype with the functional connectivity in the DLPFC (maximum MNI coordinates in right DLPFC 52, 6, 38; Z = 3.01; p_cor_ = 0.380). What was initially interpreted as a reasonable association of genetic risk with interindividual variability in the time-domain – the functional connectivity parameter – is rather a statistical artefact resulting from interindividual and between group variability in the spatial domain – the localization of seed voxels. Due to the “autocorrelation” component present in the current seed region connectivity analyses, each connectivity map obtained on the individual subject level contains strongest signals in voxels close to the seed, even at homologous voxels in the contralateral hemisphere. Thus, if there is between subject variance in seed region localization, this variance will contaminate the results of the random-effects analysis of functional connectivity, e.g. the comparison of subjects at different rs1006737 genotype. We therefore strongly recommend controlling for between subject variance of seed voxel localization by including e.g. MNI coordinates in the random-effects analyses. Further, including seed voxel coordinates in the random-effects analysis should by no means depend on statistical significant differences of seed localizations between groups. The magnitude and spatial extent of the “autocorrelation” effect is unclear, and may substantially vary between subjects, experimental paradigm, brain region, and the technical implementation of the functional connectivity analysis. For example, small average differences in the space of the seed voxels might translate into large effects with regards to the functional connectivity parameter in a specific voxel if the “autocorrelation” effect is peaked and regional specific. Loosing three degrees of freedom should be acceptable in order to obtain more valid interpretations of the functional connectivity results especially with the larger samples of subjects typically examined in imaging genetic studies.

### General Discussion

The results of functional connectivity analyses depend on a number of freely selectable technical parameters [6], [8]. Using the examples of seed voxel selection, noise regression and seed voxel localization as covariate on the second level, in the present report we demonstrated that the choice of these parameters strongly influences the interpretation of genetic effects on the neural systems' functional integration. Based on the present results, we can give three recommendations which should be considered in future connectivity analyses in this field: (i) provide detailed information about the extraction and spatial distribution of the seed voxels, (ii) if there is variability in seed voxel localization always, even without statistically significant differences between groups, introduce seed voxel coordinates as covariates on the second level, (iii) carefully interpret the direction and strength of obtained connectivity parameters.

With the present work, we did not intend to present a general and comprehensive overview of pitfalls in functional connectivity analyses. We rather wanted to show, as a proof-of-principle, how strong the assessment of genetic control over the neural systems' connectivity features depends on the technical implementation of the data analysis. For other genetic risk variants and for other tasks the effects of the described analysis parameters very likely are qualitatively and quantitatively different. However, for the purpose of the present analysis, we believe that it is not important which risk variant or which task is chosen, but to show that variability caused by different implementations of the functional connectivity analyses per se has considerable influence on the significance and the nature of described effects. While there is clear support for the choice of some parameters (e.g. the selection of seed voxels using a next local maximum approach or the correction for variability of seed voxel coordinates in a second level model), for other parameters (e.g. noise correction) an unambiguous choice is most of the time not possible. It should be noted, that fMRI data is usually analyzed for a broad range of parameters but usually only few results make it into publication. However, more information should be presented on the stability of effects with variation of technical parameters. A systematic assessment of the impact of different implementations of functional connectivity analyses, however, is practically only possible on an automated level. We therefore developed a prototype of an easy to use toolbox based on the SPM software package that allows for systematic assessment of the different above suggested implementations of seed voxel connectivity analyses. This toolbox is freely available on request.

The impact of arbitrary selectable technical parameters during the data analysis of associations of genetic variability with variability in the neural systems' functional integration is especially important in the context of replication studies. Although the application of functional connectivity methods without doubt yielded promising insights on the neural mechanisms linked to genetic variation, many of the results still have to be replicated in independent samples. High fidelity replication, however, requires precise knowledge on how the imaging data was processed and how stable the obtained results were with regard to technical variations during the data analysis. In the long run, meta-analyses can offer the appropriate tools to combine effects of risk-genes on brain connectivity across different studies and independent samples and to identify those results that are concordant or discordant with others [19]. Pooling statistical results from different studies, however, again requires detailed information about how the data was processed. It is crucial to combine imaging results that were analyzed in a similar way, not to combine statistical significant results obtained by different preprocessing strategies. In conclusion, seed-voxel functional connectivity analyses are powerful tools to assess the impact of genetic variation on the neural connectivity of brain function. For a lasting impact on the field it is important to prove the stability of their results over a range of methodological parameters.
